# Urinary neutrophil gelatinase-associated lipocalin (NGAL) can potentially predict vascular complications and reliably risk stratify patients with peripheral arterial disease

**DOI:** 10.1038/s41598-022-12286-2

**Published:** 2022-05-18

**Authors:** Mehroz Ehsan, Muzammil H. Syed, Abdelrahman Zamzam, Niousha Jahanpour, Krishna K. Singh, Rawand Abdin, Mohammad Qadura

**Affiliations:** 1 Division of Vascular Surgery, St. Michael’s Hospital, Toronto, ON M5B 1W8 Canada; 2 Department of Medical Biophysics, Schulich School of Medicine and Dentistry, University of Western Ontario, London, ON N6A 5C1 Canada; 3 Department of Medicine, McMaster University, Hamilton, ON L8S 4K1 Canada; 4 Department of Surgery, University of Toronto, Toronto, ON M5S 1A1 Canada; 5 Keenan Research Centre for Biomedical Science, Li Ka Shing Knowledge Institute, St. Michaels Hospital, Toronto, ON M5B 1W8 Canada; 6 Schulich School of Medicine and Dentistry, University of Western Ontario, London, ON Canada

**Keywords:** Biomarkers, Peripheral vascular disease

## Abstract

Neutrophil gelatinase-associated lipocalin (NGAL) is expressed in atherosclerotic plaques and implicated in the development of cardiovascular diseases*.* Peripheral arterial disease (PAD) is an atherosclerotic disease that often results in major cardiovascular events. This study aimed to prospectively examine the potential of urine NGAL (uNGAL) in predicting worsening PAD status and major adverse limb events (MALE). Baseline urine NGAL (uNGAL) and urine creatinine (uCr) concentrations were measured in PAD (*n* = 121) and non-PAD (*n* = 77) patients. Levels of uNGAL were normalized for urine creatinine (uNGAL/uCr). Outcomes included worsening PAD status, which was defined as a drop in ankle brachial index (ABI) > 0.15, and major adverse limb events (MALE), which was defined as a need for surgical revascularization or amputations. PAD patients had 2.30-fold higher levels of uNGAL/uCr [median (IQR) 31.8 (17.0–62.5) μg/g] in comparison to non-PAD patients [median (IQR) 73.3 (37.5–154.7) μg/g] (*P* = 0.011). Multivariate cox analysis showed that uNGAL/uCr levels were independently associated with predicting worsening PAD status and MALE outcomes. Cumulative survival analysis, over follow up period, demonstrated a direct correlation between elevated uNGAL/uCr levels and PAD disease progression and MALE outcomes. These data demonstrate an association between elevated uNGAL/uCr levels and worsening PAD disease status and MALE outcomes, indicating its potential for risk-stratification of PAD patients.

## Introduction

Peripheral artery disease (PAD) is a chronic atherosclerotic disease that affects the peripheral extremities^1^. Over 200 million individuals worldwide are affected by PAD; however, a substantial portion of patients are inadequately diagnosed and managed, resulting in severe complications such as lower extremity ischemia, cardiovascular events (e.g. stroke, myocardial infarction) and mortality^2–6^.

The clinical diagnosis for PAD is challenging as most patients are asymptomatic and mortality and cardiovascular outcomes are not much different in asymptomatic or symptomatic patients^7^. Therefore, identifying high risk patients, who will suffer from secondary cardiovascular complications is a challenge. Currently patients are screened for PAD using ankle-brachial index (ABI) index, the ratio of the ankle blood pressure to the highest brachial systolic pressure^8^. An ABI of ≤ 0.90 has been shown to be highly sensitive and specific, however it is not used widely. Subsequently, up to ~ 20% of PAD patients progress in their disease state to chronic limb threatening ischemia (CLTI), the most advanced severe form of the disease^9–12^. However, patients and risk factors associated with PAD disease progression are yet to be adequately identified. Due the absence of reliable prognostic risk-stratifications tools, there is a critical need for a biomarker that allows for early prediction of outcomes and stratification of disease population according to risk. Having a valid risk-stratification method would enable monitoring of high-risk patients, timely interventions, and prevent severe and costly complications.

Neutrophil gelatinase-associated lipocalin (NGAL) also known as human neutrophil lipocalin or lipocalin 2, is a 25-KDa protein, which is synthesized in the bone marrow during myelopoiesis and then stored in granules in neutrophils^13^. It is also expressed in low concentrations in several human tissues, including kidneys, lungs, stomach, and colon^14^. NGAL has been studied extensively as a biomarker for acute kidney injury^15,16^ and is shown to be upregulated in response to inflammatory stimuli such as lipopolysaccharides and IL1β^17,18^. Recent studies have highlighted its’ role in development and progression of cardiometabolic diseases^19^. NGAL has been shown to be expressed in cardiomyocytes and atherosclerotic plaques in both mice and humans^20^. Elevated plasma NGAL levels are reported to correlate with severity of coronary artery disease^21^. Elevated serum NGAL levels are also observed in obese and diabetic patients and correlate with C-reactive protein levels^22^.

Although elevated NGAL levels have been associated with cardiovascular disease, the levels of NGAL, especially urinary NGAL (uNGAL), has not yet been investigated in PAD patients. In this study, we sought to assess the relation between baseline uNGAL concentrations and patients with PAD. Furthermore, we aimed to investigate its prognostic value in determining PAD-related complications including worsening PAD status and major adverse limb events.

## Results

### Cohort description and characteristics

Demographic data and clinical characteristics of all 198 patients which comprised of 121 PAD patients (61%) and 77 non-PAD patients (39%) are presented in Table 1. Overall, the mean age of the cohort was 66 ± 11 years. There were 128 male participants (65%), 135 patients (68%) with hypertension, 147 patients (74%) with hypercholesteremia, 49 patients (25%) with diabetes, and 161 patients (81%) who were current or past smokers. The mean age, percentage of patients with hypertension, hypercholesteremia, diabetes, smoking history, and history of coronary artery disease were significantly higher in the PAD group than in the non-PAD group. As expected, no significant change was found in the mean glomerular filtration rate (GFR) between PAD and non-PAD patients (67 ± 10 vs. 63 ± 13, *P* = 0.09). Overall, the median (IQR) uNGAL/uCr level was 48.7 (23.7–110.4). The median (IQR) uNGAL/uCr concentration in PAD group [73.3 (37.5–154.7) μg/g] was 2.30-times higher (*P* = 0.011) than non-PAD group [31.8 (17.0–62.5) μg/g].

**Table 1:** Baseline clinical and laboratory characteristics.

Demographics and clinical characteristics (at baseline)	Overall (*n* = 198)	non-PAD (*n* = 77)	PAD (*n* = 121)	*P*^α^
**Mean (SD)**^**‡**^				
ABI	0.83 (0.26)	0.97 (0.21)	0.64 (0.20)	**0.001**
Age, years	66 (11)	63 (13)	67 (10)	**0.008**
GFR	90.6 (18.3)	93.4 (17.4)	88.9 (18.7)	0.091
**N** **(%)**^**¶**^				
Sex, male	128 (65)	50 (65)	78 (65)	0.946
Hypertension	135 (68)	46 (60)	89 (74)	**0.042**
Hypercholesteremia	147 (74)	44 (57)	103 (85)	**0.001**
Diabetes	49 (25)	12 (16)	37 (31)	**0.017**
Smoking	161 (81)	54 (70)	107 (88)	**0.001**
History of congestive heart failure	5 (3)	1 (1)	4 (3)	0.395
History of coronary artery disease	59 (30)	14 (18)	45 (37)	**0.004**

Frequencies and percentages were calculated for categorical variables; all numbers were rounded up with zero decimal place.

All *p*-values were rounded to three decimal places, *p* < 0.05 in bold.

ABI: Ankle Brachial Index.

GFR: Glomerular Filtration Rate.

^α^The significance of the difference between PAD and non-PAD groups.

^‡^Compared using student’s t-test.

^**¶**^Compared using chi-square test.

### Clinical outcomes at two years

All patients were prospectively followed for a two-year period (refer to Table 2 for clinical outcomes). Follow-up data was available for 94.8% of patients, with a mean duration of 21.4 ± 3.4 months. During the two-year follow-up, we noted that 39 patients (20%) had an ABI drop of ≥ 0.15, 30 patients (15%) required vascular interventions, 5 patients (3%) underwent major limb amputation, and 34 (17%) patients had major adverse limb events (MALE). The rate of change of ABI, number of vascular interventions, and MALE outcomes were significantly higher in PAD group compared to non-PAD group (Table 2). Some non-PAD patients experienced a ABI drop (≥ 0.15), however their ABI remained within the normal range (0.9–1.3) and therefore were not categorized as having PAD.

**Table 2:** Distribution of adverse events in PAD patients compared to non-PAD patients during 2-year follow-up.

Event	Overall (*n* = 198) *N* (%)	Non-PAD (*n* = 77) *N* (%)	PAD (*n* = 121) *N* (%)	P*
ABI ≥ -0.15	39 (20)	7 (9)	32 (26)	**0.005**
Vascular intervention	30 (15)	0 (0)	30 (25)	**0.001**
Major limb amputation	5 (3)	0 (0)	5 (4)	0.062
MALE	34 (17)	0 (0)	34 (28)	**0.001**

*The significance of the difference between non-PAD group and PAD group using chi-square test.

All *p*-values were rounded to three decimal places, *P* < 0.05 in bold.

ABI: Ankle Brachial Index; MALE—defined as vascular intervention or limb loss.

### Risk stratification of patients based on their uNGAL/uCr concentrations

Based on the median uNGAL/uCr value of 48.7 μg/g as the cutoff point, patients were divided into 2 groups: (1) Low NGAL group (*n* = 99): uNGAL/uCr ≤ 48.7 μg/g, and (2) high NGAL group (*n* = 99): uNGAL/uCr > 48.7 μg/g. The characteristics of these 2 groups are presented in Table 3. The only significant difference in comorbidities between the groups at baseline was the percentage of PAD patients, which was higher in the high NGAL group (77%, *P* = 0.001). Relative to low NGAL group, our data demonstrates that patients within the high NGAL group had a higher incidence of ABI ≥ -0.15 (11% vs. 28%, *P* = 0.002), need for vascular intervention (10% vs. 20%, *P* = 0.041) and MALE outcomes (12% vs. 22%, *P* = 0.044). However, there was no significant difference between incidence of major limb amputation between low and high NGAL groups (2% vs. 3%, *P* = 0.651).

**Table 3:** Baseline patient demographics and clinical characteristics of the Low NGAL and High NGAL study subgroups.

Demographics and clinical characteristics (at baseline)	Low NGAL (*n* = 99)	High NGAL (*n* = 99)	*P*-value
**Mean (SD)**^**‡**^			
Age, years	66 (11)	67 (11)	0.565
GFR	92.8 (19.1)	88.5 (17.3)	0.093
***N* (%)**^**¶**^			
Peripheral artery disease	45 (46)	76 (77)	**0.001**
Sex, male	62 (63)	59 (60)	0.071
Hypertension	68 (69)	67 (68)	0.879
Hypercholesteremia	72 (73)	75 (76)	0.626
Diabetes	23 (23)	29 (29)	0.138
Smoking, current + past	76 (77)	85 (86)	0.101
History of coronary artery disease	31 (31)	28 (28)	0.641
**Event rate at 2 years** ***N* (%)**^**¶**^			
ABI ≥ -0.15	11 (11)	28 (28)	**0.002**
Vascular intervention	10 (10)	20 (20)	**0.041**
Major limb amputation	2 (2)	3 (3)	0.651
MALE	12 (12)	22 (22)	**0.044**

Low NGAL group (*n* = 99): NGAL ≤ 48.7 μg/g.

High NGAL group (*n* = 99): NGAL > 48.7 μg/g.

eGFR: estimated Glomerular Filtration Rate.

^‡^Compared using student’s t-test.

^**¶**^Compared using chi-square test.

ABI: Ankle Brachial Index; MALE—defined as vascular intervention or limb loss.

Significant values are in bold.

### Time series analysis and adverse PAD complications

**Figure 1:**
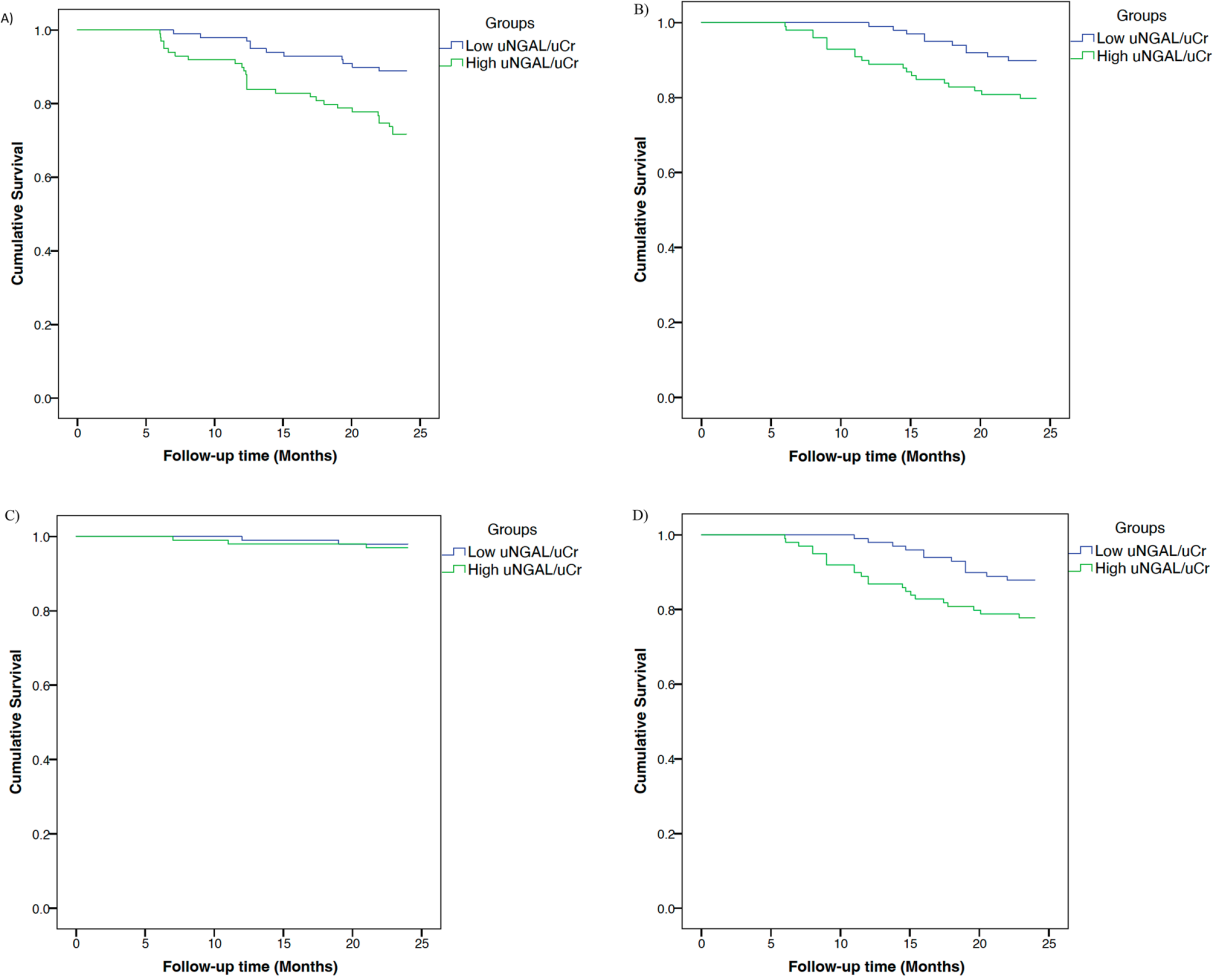
Kaplan–Meier analysis of PAD-related complications event-free survival in High uNGAL and Low uNGAL subgroups. Kaplan Meier estimate for event-free survival, including (**A**) ABI ≥  − 0.15, (**B**) vascular intervention, (**C**) major limb amputation, and (**D**) MALE, for Low NGAL group (*n* = 99, uNGAL/uCr ≤ 48.7 μg/g) compared to High NGAL (n = 99, uNGAL/uCr NGAL > 48.7 μg/g) based on the median uNGAL/uCr values. MALE = vascular Intervention and major limb amputation.

There was a statistically significant difference in PAD-related complications time series analysis between those who had high uNGAL/uCr and low uNGAL/uCr concentrations. Kaplan–Meier analysis demonstrated that elevated levels of uNGAL/uCr (≥ 48.7 μg/g) could reliably risk stratify patients for ABI ≥ -0.15 (*P* = 0.002; log rank = 9.44), need for vascular intervention (*P* = 0.037; log rank = 4.34) and MALE (*P* = 0.044; log rank = 4.05). However, there was no significant difference in event-free survival rate for major limb amputation between low and high uNGAL/uCr groups (*P* = 0.648; log rank = 0.208) (Fig. 1*A-D*). Event-free survival rates for change in ABI ≥ −0.15 at 1 year and 2 years were 97% and 89% in the low uNGAL/uCr group and 90% and 72% in the high uNGAL/uCr group. Moreover, event-free rates for vascular intervention at 1-year and 2-years were 99% and 90%, respectively in the low uNGAL/uCr group and 89% and 80%, respectively in the high uNGAL/uCr group. Finally, MALE-free survival rates at 1-year and 2-years were 98% and 88%, respectively in the low uNGAL/uCr group and 87% and 78%, respectively in the high uNGAL/uCr group.

### Cox regression analysis of uNGAL/uCr levels and PAD related complications

The ability of uNGAL/uCr to predict PAD-related complications (worsening PAD status and MALE outcomes) was examined by univariate and multivariate Cox proportional hazard analyses. Univariate analysis showed that for each unit increase in uNGAL/uCr, there was an association with subsequent ABI drop ≥ 0.15 (HR 2.33, 95% CI 1.42–3.83), need for vascular intervention (HR 1.78, 95% CI 1.03–4.11, and MALE (HR 1.47, 95% CI 1.05–2.06) (Table 4). Multivariate logistic regression analysis showed that, despite adjusting for age, sex, GFR, hypertension, hypercholesteremia, smoking, diabetes, and history of CAD, uNGAL/uCr level remained an independent determinant for ABI ≥ -0.15 (HR 2.37, 95% CI 1.52–3.72), need for vascular intervention (HR 1.82, 95% CI 1.06–3.25) and MALE outcomes (HR 1.49, 95% CI 1.09–2.11). Although we observed higher incidences of major limb amputation in patients with PAD, we did not observe a statistically significant hazard ratio for this event.

**Table 4:** Cox regression models for adverse events in PAD patients compared to non-PAD patients for one unit increase of log (uNGAL/uCr).

Event	Unadjusted HR (95% CI)	*P*-value	Adjusted HR (95% CI) ^‡^	*P*-value
ABI ≥ -0.15	*2.33 (1.42–3.82)*	**0.001**	*2.37 (1.52–3.72)*	**0.001**
Vascular intervention	*1.78 (1.03–4.11)*	**0.032**	*1.82 (1.06–3.25)*	**0.017**
Major limb amputation	1.25 (0.25–3.28)	0.789	1.26 (0.23–4.79)	0.594
MALE	1.47 (1.05–2.06)	**0.025**	1.49 (1.09–2.11)	**0.011**

^‡^Adjusted for age, sex, eGFR, hypertension, hypercholesteremia, smoking, diabetes, history of CAD.

All *p*-values were rounded to three decimal places, *P* < 0.05 in bold.

ABI: Ankle Brachial Index; MALE—defined as vascular intervention or limb loss.

Significant values are in italic.

## Discussion

In this prospective study of consecutive patients, we evaluated the utility of uNGAL/uCr as a predictor of PAD-related complications over a period of 2 years. Our findings show that uNGAL/uCr levels are elevated in PAD patients and predict PAD-related complications. Patients with high levels of uNGAL/uCr exhibited higher rates of worsening PAD status, MALE outcomes and need for vascular intervention compared to patients with low levels of uNGAL/uCr. Moreover, our findings demonstrated that elevated uNGAL/uCr levels could assist in stratifying patients at risk of poorer PAD prognosis.

NGAL was initially identified as an inflammatory marker which is released form neutrophils in response to bacterial infection^13^. NGAL is highly expressed in kidney tubule cells and its levels in urine rise before serum creatinine levels change hence it being a marker of acute kidney injury (AKI)^15,16^. There has been increasing evidence shows that NGAL levels correlate with cardiovascular diseases progression and outcomes. NGAL is expressed in cardiomyocytes, and endothelial cells, smooth muscle cells, and macrophages of atherosclerotic plaques^23–26^. Elevated circulatory NGAL levels independently predict all-cause mortality and MACE in ST-segment elevation myocardial infarction patients following primary percutaneous coronary intervention^24^, correlate with atherosclerotic burden and predict predicts all-cause mortality in coronary artery patients^23–26^, and are an independent predictor of 3-month risk of death and 6-month risk of readmission in heart failure patients^27,28^. Elevated uNGAL on the first day of admission is a strong prognostic factor for poor long-term outcomes in acute decompensated heart failure patients^29^. Consistent with these findings our data suggest that uNGAL levels are associated with worse outcomes in PAD patients and perhaps NGAL is released into circulation increasingly with worsening PAD status and subsequently cleared by kidney. Very few studies have investigated the predictive prognostic value of NGAL, especially uNGAL, and especially in context of long-term outcomes in PAD patients. In addition to its association with long term outcomes uNGAL also improved predictive accuracy of PAD-related adverse events, prompting that patients with elevated urinary NGAL may warrant closer monitoring for adverse events. The goal of our study was to establish a marker for PAD independent of the traditional ABI measurement which is measured unreliably in the community. We stratified the population by uNGAL/uCR to highlight that normalized uNGAL levels can be used to predict outcomes in these patients. Though the outcomes in the high uNGAL/uCR are almost all in PAD group, these analysis within high and low uNGAL/uCR show the importance of this potential biomarker on its own and allow us to work towards establishing a clinically significant cutoff in future studies.

The increased NGAL expression in atherosclerotic disease is believed to be due to its interaction with metal metalloproteinase 9 (MMP-9). Increased MMP-9 levels and activity are well established in arterial plaque architecture. NGAL binds to MMP-9 inhibiting its degradation leading to higher and prolonged activity^26^. The resulting tissue remodeling contributes to plaque instability and eventually leads to the development of acute coronary syndrome^30^. Circulating levels of MMP-9 are also known to be elevated in PAD patients with intermittent claudication and critical ischemia^30,31^.

The study has a few limitations. Firstly, the current findings are based on patients recruited from a single-center with a relatively small sample size, which may limit certain statistical analyses (e.g. matching) as well as generalizability of these results. The patient population in this study is predominantly male which may not be representative of the current prevalence of PAD, however, it is not unusual to have higher proportion of male study subjects in PAD studies^32,33^. Secondly, there is a lack of mechanistic understanding underlying the increase in uNGAL in PAD, and therefore, we cannot ascertain whether NGAL levels are driving the disease and its outcomes or is an irrelevant bystander in progression of PAD. This latter notion is supported by the fact that plasma NGAL levels are elevated in inflammation, a hallmark of PAD. Thirdly, the study did not evaluate certain inflammatory markers such as C-reactive protein and therefore would aim to include them in further studies Further, prospective longitudinal studies with larger sample sizes and multiple uNGAL measurements would allow us to match cohorts and highlight the predictive value of uNGAL as a biomarker; however, the current findings serve as a strong foundation for future studies investigating NGAL as a biomarker for PAD.

A key message of our findings is that uNGAL/uCr has the potential of being a good biomarker for predicting PAD-related complications. This is clinically significant as NGAL levels have been shown to correlate with atherosclerotic disease. Based on these findings, it is also plausible to suggest that uNGAL/uCr can be used to risk stratify patients with PAD at early stage of disease, however further study in larger cohorts is necessary to validate this suggestion.

## Methods

### Ethics approval

The Research Ethics Board at St. Michael’s Hospital, University of Toronto approved the study (#16–375, 8 February 2017) and written informed consents were obtained from all participants. All methods were performed in accordance with the relevant guidelines and regulations.

### Patient recruitment and assessment

For the study consecutive 198 patients (77 non-PAD and 121 PAD patients) were recruited between August 2018 and August 2019 from the ambulatory vascular surgery clinics at St. Michael’s Hospital, Toronto. Patients were followed for a total of 24 months with follow up visits scheduled at 12 months and 24 months from the time of initial assessment. As previously described, patients underwent a complete history and physical exam, in addition to a lower limb arterial ultrasound to measure ABI^34–36^. ABI measurements were taken by two experienced vascular technologists at a credited vascular laboratory at St. Michael’s Hospital. Both vascular technologists rigorously followed the ABI measurement protocol recommended by the American Heart Association and the Society of Vascular Surgery clinical guidelines^37,38^. In this study, patients with an ABI value of < 0.9, in addition to lack of posterior tibial and dorsalis pulses in at least one leg, with or without claudication, were defined as PAD patients. Patients presenting with a normal arterial ultrasound, an ABI ≥ 0.9, presence of palpable distal pulses, and no clinical history of claudication were defined as control or non-PAD group. In situations where the ABI could not be accurately determined due to non-compressible tibial vessels, toe brachial index (TBI) measurements were performed. As per ESC/ESVM guidelines, patients with TBI < 0.7 were identified as PAD group and patients with a TBI ≥ 0.7 were identified as control or non-PAD group^8,39^.

Patients with a history of chronic kidney disease (stages 3–5 as per Kidney Disease Outcomes Quality Initiative 2002 guidelines—defined as having an estimated glomerular filtration rate of less than 60 mL/min/1.73 m^2^) were excluded. We also excluded patients with active infection, diagnosis of sepsis or malignancy.

### Baseline measurements and sample collection

Baseline medical history including demographics, history of cardiovascular diseases, cardiovascular risk factors (hypertension, hypercholesterolemia, and diabetes), and smoking status was collected from all patients as described previously^34–36^. Urinary analyses were performed on mid-stream urinary samples, which were stored at −80 °C on the day of collection and thawed on slowly on ice.

### Urinary NGAL multiplex assay

To determine the concentrations of NGAL levels in urine, samples were examined in duplicate using MILLIPLEX® Mouse Kidney Injury Magnetic Bead Panel 2 (EMD-Millipore; Billerica, MA) on a calibrated MagPix analyzer (Luminex Corp; Austin, Texas). To minimize any inter-assay variability, all analyses were carried out on the same day. Sample intra-assay and inter-assay coefficient of variability (CV) was < 10%. At least 50 beads for uNGAL were acquired using Luminex xPonent software and analyzed using Milliplex Analyst software (v.5.1; EMD-Millipore).

### Measurement of urinary creatinine and normalization of UNGAL

As described previously, uNGAL was normalized with urinary creatinine (uCr) to adjust for the potential influence of urinary concentration and different hydration status on single-spot urine samples, to achieve normalized uNGAL/uCr (μg/g)^34^. uCR levels which were measured at the Core Laboratory at St. Michael’s Hospital using the Beckman Coulter AU680 laboratory analyzer (Beckman Coulter; Pasadena, California).

### Follow-up and measured outcomes

Follow-up visits were performed at 12-months as well as at the end of the study. ABI values, PAD symptomatic status, PAD-related interventions (i.e. revascularization or amputation), and any changes made to concomitant treatment were recorded at these outpatient visits. Self and EMR reported PAD-related hospitalization and emergency room visits were also recorded during these visits. The primary outcome of this study was incidence of worsening PAD status in patients during the follow-up period, defined as change in ABI ≥ −0.15 from baseline. The cutoff value of change in ABI is well established in previous studies^40–42^. Secondary outcome included the incidence of major adverse limb events (MALE)—defined as composite of PAD-specific outcomes such as severe limb ischemia leading to an arterial intervention (open or endovascular revascularization) or major vascular amputation (above the level of the ankle).

### Statistical analysis

Demographics and clinical characteristics were expressed as mean and standard deviations (SDs), or percentages (%). Continuous data were compared using Student’s t-test if conforming to normal distribution, otherwise Mann–Whitney U test was used. Chi-square test was used for categorical variables comparisons. Normalized uNGAL/uCr levels were not normally distributed, as determined by the Kolmogorov–Smirnov test, and were logarithmically transformed before survival analysis and expressed as medians with inter quartile ranges (IQRs).

Event rates at 24 months were calculated for drop in ABI (≥ 0.15), MALE outcomes, or individual components of MALE (need for revascularization, need for major amputation). For the Kaplan–Meier curves, median of uNGAL/uCr levels were used as cutoff points. The event-free curves were computed, and comparison of event-free survival between sub-groups were performed using the log-rank test. Statistical significance was established at *P* < 0.05 (2-sided). A Cox proportional hazard analysis was performed to determine uNGAL/uCr predictability for change in ABI (≥−0.15), MALE, and of MALE outcomes for the entire study cohort. Variables that were deemed confounders [age (in years), sex (male vs female), GFR (mL/min), hypertension (yes vs no), hypercholesteremia (yes vs no), smoking (yes vs no), diabetes (yes vs no), and history of CAD (yes vs no)] were entered into the multivariate analysis. SPSS software, version 23 (SPSS Inc., Chicago, Illinois, USA) was used for data entry and statistical analysis. Microsoft excel was used for graphical illustrations.

## Data Availability

All data generated or analysed during this study are included in this published article.
